# Adverse cardiovascular and kidney outcomes in people with SARS-CoV-2 treated with SGLT2 inhibitors

**DOI:** 10.1038/s43856-024-00599-4

**Published:** 2024-09-11

**Authors:** Taeyoung Choi, Yan Xie, Ziyad Al-Aly

**Affiliations:** 1 Clinical Epidemiology Center, Research and Development Service, VA Saint Louis Health Care System, Saint Louis, MO USA; 2 Veterans Research and Education Foundation of Saint Louis, Saint Louis, MO USA; 3 Division of Pharmacoepidemiology, Clinical Epidemiology Center, Research and Development Service, VA Saint Louis Health Care System, Saint Louis, MO USA; 4 Department of Medicine, Washington University School of Medicine, Saint Louis, MO USA; 5 Nephrology Section, Medicine Service, VA Saint Louis Health Care System, Saint Louis, MO USA; 6 Institute for Public Health, Washington University in Saint Louis, Saint Louis, MO USA

**Keywords:** SARS-CoV-2, Cardiology, Kidney diseases, Diabetes, Viral infection

## Abstract

**Background:**

Whether use of SGLT2 inhibitors reduces the risk of cardiovascular and kidney events in people who contracted SARS-CoV-2 infection is not clear.

**Methods:**

We used the healthcare databases of the United States Department of Veterans Affairs to build a cohort of 107,776 participants on antihyperglycemic therapy and had SARS-CoV-2 infection between March 01, 2020 and June 10, 2023. Within them, 11,588 used SGLT2 inhibitors and 96,188 used other antihyperglycemics. We examined the risks of major adverse cardiovascular events (MACE)—a composite of death, myocardial infarction and stroke, and major adverse kidney events (MAKE)—a composite of death, eGFR decline > 50%, and end stage kidney disease after balancing baseline characteristics between groups through inverse probability weighting. Survival analyses were conducted to generate hazard ratio (HR) and absolute risk reduction per 100 person-years (ARR).

**Results:**

Over a median follow up of 1.57 (IQR: 1.05–2.49) years, compared to the control group, SGLT2 inhibitors use is associated with reduced risk of MACE (HR 0.82 (0.77, 0.88), ARR 1.73 (1.21, 2.25)) and reduced risk of MAKE (HR 0.75 (0.71, 0.80), ARR 2.62 (2.13, 3.11)). Compared to the control group, SGLT2 inhibitors use is associated with reduced risk of the secondary outcomes of hospitalization (HR 0.94 (0.90, 0.98), ARR 1.06 (1.36, 1.76)), anemia (HR 0.71 (0.65, 0.76), ARR 2.43 (1.95, 2.90)), and acute kidney injury (HR 0.84 (0.79, 0.89), ARR 1.86 (1.29, 2.42)).

**Conclusions:**

Among people with SARS-CoV-2 infection on antihyperglycemic therapy, compared to those on other antihyperglycemics, those on SGLT2 inhibitors have less risk of adverse cardiovascular and kidney outcomes.

## Plain language summary

SARS-CoV-2 infection leads to significant increase in risk of heart and kidney problems both shortly after infection and in the long-term. In this study, we evaluated whether SGLT2 inhibitors could reduce the risk of major adverse heart and kidney events in people with SARS-CoV-2 infection. SGLT2 inhibitors are a type of medication used to treat diabetes by lowering the amount of sugar in the blood. We compared a large group of people during and after SARS-CoV-2 infection and found that those who were using SGLT2 inhibitors had less major adverse heart and kidney problems than those who were using other types of sugar-lowering medications. Our findings could be useful for optimizing approaches to reduce risk of heart and kidney problems among people with diabetes and SARS-CoV-2 infection.

## Abstract (web summary)

Choi et al. report on the effectiveness of SGLT2 inhibitors in reducing risk of major adverse cardiovascular events (MACE) and major adverse kidney events (MAKE) in people with SARS-CoV-2 infection. They show that compared to other antihyperglycemics, SGLT2 inhibitors reduce the risk of both MACE and MAKE after SARS-CoV-2 infection.

## Introduction

SARS-CoV-2 infection is associated with increased risk of adverse cardiovascular and kidney outcomes in both the acute and post-acute phase of the COVID-19 illness^1–5^. SGLT2 inhibitors have been shown in multiple randomized trials to reduce the risk of major adverse cardiovascular events (MACE) (death, myocardial infarction and stroke) and major adverse kidney events (MAKE) (death, eGFR decline > 50% and end-stage kidney disease (ESKD))^6–9^. Whether use of SGLT2 inhibitors reduces the risk of cardiovascular and kidney events in people who contracted SARS-CoV-2 infection is not clear.

The DARE-19 randomized placebo-controlled trial enrolled 1250 patients with cardiometabolic risk factors who were hospitalized with COVID-19. The results of this trial showed that treatment with dapagliflozin (vs placebo) yielded a hazard ratio of 0.80 (95% CI 0.58–1.10) for the composite outcome of organ dysfunction or death; and the hazard ratio for death was 0.77 (95% CI 0.52–1.16). Both risk estimates were favorable yet imprecise and statistically non-significant—likely due to low power^10^. The RECOVERY trial reported that in 4271 adults hospitalized with COVID-19, empagliflozin was not associated with reductions in 28-day mortality, duration of hospital stay, or risk of progressing to invasive mechanical ventilation or death^11^.

Both DARE-19 and RECOVERY exclusively enrolled people who were hospitalized with COVID-19 (who do not represent the majority of people with COVID-19), examined only acute outcomes at 28 days, and did not evaluate cardiovascular or kidney outcomes.

Yet, it is now widely recognized that people with SARS-CoV-2 infection—including those who were hospitalized and non-hospitalized during the acute phase of the infection — experience increased risk of adverse cardiovascular and kidney events in the acute and post-acute phase of the disease and that the risk may remain elevated even a year after infection^3,4,12,13^.

Whether use of SGLT2 inhibitors reduces risk of adverse cardiovascular and kidney outcomes in people with SARS-CoV-2 infection is still not yet known. Addressing this question will inform prevention and treatment approaches of the adverse cardiovascular and kidney consequences of SARS-CoV-2 infection.

In this study, we used the electronic health records of the US Department of Veterans Affairs and identified 11,588 users of SGLT2 inhibitors and 96,188 users of other antihyperglycemics who had SARS-CoV-2 between March 01, 2020 and June 10, 2023. We then applied inverse probability weighting to balance the health and demographic characteristics between antihyperglycemics users who received SGLT2 inhibitors vs those who did not (the control group) and evaluated whether treatment with SGLT2 inhibitors was associated with reduced risk of MACE (defined as composite of death, myocardial infarction and stroke) and MAKE (defined as composite of death, eGFR decline > 50%, and end stage kidney disease (ESKD)). In this study, we find that among people with SARS-CoV-2 infection on antihyperglycemic therapy, compared to those on other antihyperglycemics, those on SGLT2 inhibitors have less risk of MACE (Hazard ratio (HR) 0.82 (0.77, 0.88), absolute risk reduction per 100 person-years (ARR)1.73 (1.21, 2.25)) and MAKE (HR 0.75 (0.71, 0.80), ARR 2.62 (2.13, 3.11)). We conclude that among people with SARS-CoV-2 infection on antihyperglycemic therapy, compared to those on other antihyperglycemics, those on SGLT2 inhibitors have less risk of adverse cardiovascular and kidney outcomes.

## Methods

### Setting

The study was conducted using data from the US Department of Veterans Affairs (VA) healthcare databases—which operates 1293 healthcare facilities including 171 medical centers and 1112 outpatient sites. As the largest integrated healthcare system in the US, the VA provides comprehensive healthcare services to veterans of the US armed forces. These services encompass preventive and health maintenance care, outpatient and inpatient hospital care, mental healthcare, home healthcare, primary and specialty care, geriatric and extended care, as well as provision of pharmaceuticals, medical equipment and prosthetics.

### Data sources

The healthcare databases of the US Department of Veterans Affairs were utilized in this study. These databases include information collected during patients’ routine healthcare encounters and are updated daily. The data domains include outpatient and inpatient diagnoses, pharmacy, and laboratory results. Vaccination status was collected from the VA COVID-19 Shared Data Resource. The Area Deprivation Index (ADI)—a composite measure of income, education, employment, and housing—served as a summary measure of contextual disadvantage at the participants’ residential locations^14^.

### Cohort

**Fig. 1:**
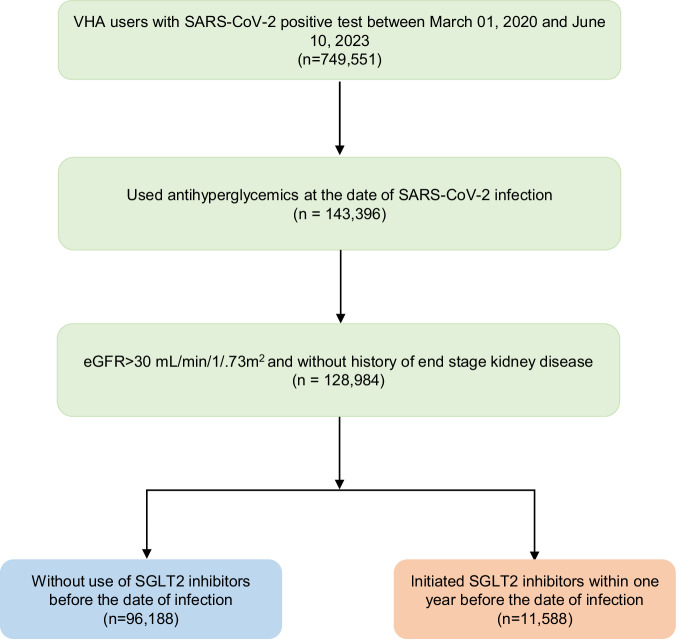
Cohort flowchart. Cohort construction flow chart.

We present a flowchart of cohort construction in Fig. 1. We enrolled 749,551 users of VA health care system who had a positive SARS-CoV-2 test result between March 01, 2020 and June 10, 2023. The date of first positive test was set to be T_0_. We further selected participants who used antihyperglycemics at the date of infection based on prescription records (N = 143,396). We then removed participants who had end stage kidney disease or had an eGFR < 30 mL/min/1.73m^2^ (N = 128,984). Participants with incident use of SGLT2 inhibitor within one year before T_0_ were selected into the SGLT2 inhibitors group (N = 11,588) and participants without use of SGLT2 inhibitors before T_0_ were selected into the control group (N = 96,188). Participants were followed until July 10, 2023.

### Exposure

The exposure group was defined as participants who initiated SGLT2 inhibitors within 1 year prior to the date of infection and continued using SGLT2 inhibitors at the date of infection. The control group comprised participants without a history of SGLT2 inhibitors use, and were using any other antihyperglycemic besides SGLT2 inhibitors at the date of infection.

### Outcomes

In this study, we evaluated the risk of MACE, defined as a composite of death, myocardial infarction and stroke; and also risk of MAKE, defined as a composite of death, eGFR decline > 50%, and ESKD^15–17^. We also evaluated the risk of secondary outcomes, including each individual component of the composite outcomes (death, stroke, myocardial infarction, eGFR decline > 50%, and ESKD), along with hospitalization, anemia, and acute kidney injury (Supplementary Table 1). The risk of incident outcomes was assessed within participants who had no history of the outcome within the 3 years preceding T_0_.

### Covariates

Baseline covariates that may affect the exposure and the outcomes were ascertained within three years before T_0_ based on literature review and prior knowledge^1,3,15,18–32^, where covariates status closest to T_0_ were used. We selected demographic factors including age, self-declared race (white, Black, and other), self-declared sex, ADI, health factors including COVID-19 vaccine status (unvaccinated, received 1 or 2 doses vaccine, boosted), body mass index (BMI), smoking status (never, former, and current), and use of long-term care. We also selected covariates representing comorbidities including eGFR, low-density lipoprotein cholesterol (LDL), systolic and diastolic blood pressure, cancer, cardiovascular disease, hyperlipidemia, peripheral artery disease, chronic lung disease, dementia, acute kidney injury, acute pancreatitis, venous thromboembolism, immune dysfunction, and albuminuria. Medication use including history use of ACE/ARB, calcium channel blockers, beta blockers, diuretics, statins were also included as covariates. To adjust for antihyperglycemics use at date of infection, we also accounted for the use of metformin, DPP4 inhibitors, GLP-1RAs, sulfonylureas, thiazolidinediones, insulin and other antihyperglycemics including alpha or amylin or meglitinide at the date of infection. In addition, we adjusted for outpatient COVID-19 treatments, including COVID-19 antivirals including nirmatrelvir, molnupiravir, and remdesivir and COVID-19 monoclonal antibody medications. Healthcare utilization including number of outpatient and inpatient encounters, number of blood panel tests, number of outpatient prescriptions, number of HbA1c tests and number of Medicare outpatient and inpatient encounters and pandemic related characteristics represented by week of the T_0_ were also adjusted. All missing continuous variables (2.76% of eGFR, 3.07% of BMI, 0.08% of blood pressure and 0.65% of LDL) were imputed based on fully conditional specification method conditioning on all covariates and assigned values based on predictive mean matching^33^. Continuous variables were transformed into restricted cubic spline functions in the process of modeling^34^.

### Statistical analysis

Baseline characteristics of those on SGLT2 inhibitors and those in the control group were reported. Differences of the baseline characteristics between the two groups were assessed using absolute standardized differences where a value of less than 0.1 was considered evidence of good balance^35^.

Inverse probability weighting was used to balance the differences in baseline characteristics between SGLT2 inhibitors and the control group. Multivariate logistic regressions were constructed to estimate the probability of belonging to the SGLT2 inhibitors group (the propensity score). We then constructed the weighting toward the SGLT2 inhibitors group by assigning weights of 1 for those in the SGLT2 inhibitors group and weights of propensity score/(1-propensity score) for those in the control group^36^. Weighted Cox survival models were employed to estimate the association between SGLT2 inhibitors and outcomes. Event rates and absolute risk reductions were estimated based on the survival probabilities of the two groups generated from the survival model. To estimate the risk of incident outcomes, outcome-specific propensity score models and survival models were conducted among participants with no history of the evaluated outcome.

We further examined the risk of MACE and MAKE across various subgroups including age ( ≤ 60 and > 60 years), sex (male and female), race (white and Black), vaccination status (unvaccinated, received 1 or 2 doses of vaccine, boosted), status of hospitalization during acute phase of infection, metformin use, insulin use, cardiovascular disease, BMI ( > 30 and ≤ 30 kg/m^2^) and eGFR ( ≥ 60 and < 60 ml/min/1.73m^2^).

To examine the robustness of our findings, we conducted multiple sensitivity analyses. These included (1) applying an overlap weighting method instead of the inverse probability weighting method used in the primary approach^37^; (2) using doubly robust adjustment for covariates in the weighted survival model, instead of solely balancing based on the weighting in the primary approach^38^; (3) redefining the exposure group as those who initiated SGLT2 inhibitors within 180 days and, separately, within 90 days before the infection, to proxy incident use instead of defining the exposure group as those who initiated SGLT2 inhibitors within 1 year before infection as in the primary approach; (4) additionally adjusting for healthcare utilization factors such as the number of outpatient and inpatient visits, the number of laboratory tests, and the number of prescriptions received during follow-up, instead of only adjusting for baseline characteristics as in the primary approach; (5) additionally adjusting for time-varying HbA1c values during follow-up, instead of only adjusting for baseline HbA1c as in the primary approach; (6) conducted per-protocol analyses based on inverse probability of censoring weight where the protocol for the SGLT2 inhibitors group was defined as continued use of SGLT2 inhibitors during follow up; and the protocol for the control group, the protocol was defined as non-use of SGLT2 inhibitors during follow up, whereas the primary analyses employed intention to treat approach^39^.

In this study, 95% CI of the hazard ratio that does not cross 1 and 95% CI of the absolute risk reduction that does not cross 0 were considered statistically significant. Data management and analyses were performed with SAS Enterprise Guide, version 8.3 (SAS Institute, Cary, NC). Data visualizations were performed in R 4.2.2 (R Foundation for Statistical Computing, Vienna, Austria).

### Ethical approval

This study used data from the U.S. Department of Veterans Affairs healthcare database. This research project was reviewed and approved by the Institutional Review Board (IRB) of the VA Saint Louis Health Care System (Protocol number 1606333). The Institutional Review Board waived the need to obtain informed consent from veterans whose data is included in the healthcare database.

### Reporting summary

Further information on research design is available in the Nature Portfolio Reporting Summary linked to this article.

## Results

The study enrolled 107,776 participants who had a positive SARS-CoV-2 test. Within them, 11,588 participants were in the SGLT2 inhibitors group and 96,188 participants were in the control group of other antihyperglycemics. Baseline demographic and health characteristics of the SGLT2 inhibitors and the control groups before and after inverse probability weighting are presented in Supplementary Data 1 and Supplementary Data 2, respectively. Assessment of the absolute standardized mean differences (SMDs) of the demographic and health characteristics between the SGLT2 inhibitors and the control groups after weighting yielded SMDs below 0.1 – indicating good balance (Supplementary Data 2, Supplementary Fig. 1).

**Fig. 2:**
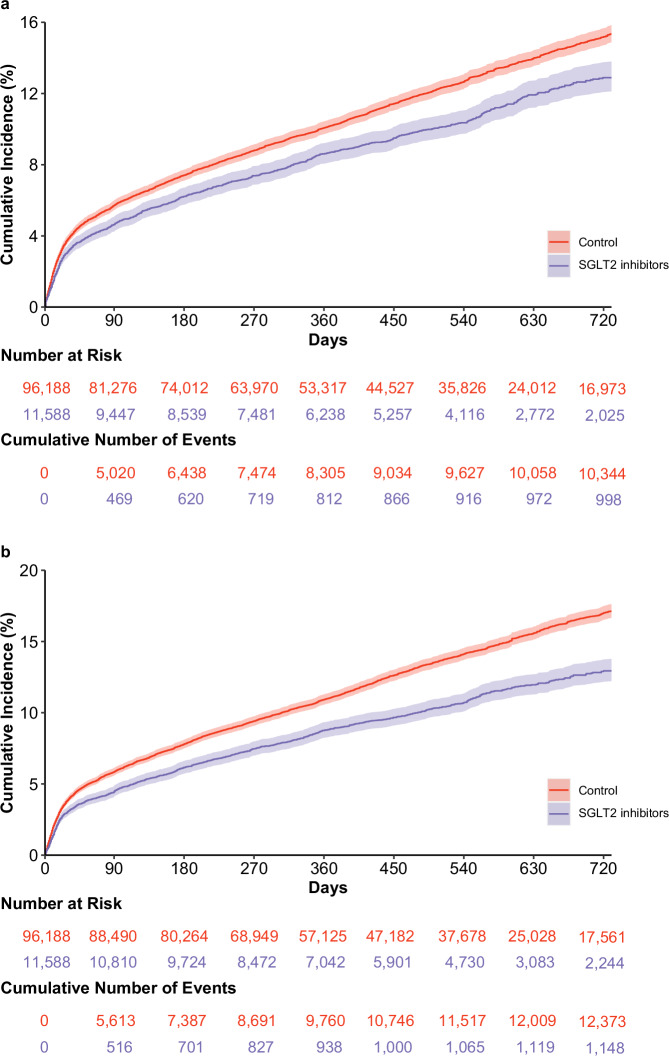
Cumulative incident function for the SGLT2 inhibitors and the control group. **a** Major adverse cardiovascular events (MACE); **b** Major adverse kidney events (MAKE). MACE was a composite of death, myocardial infarction and stroke. MAKE was a composite of death, eGFR decline > 50%, and end stage kidney disease. Cumulative incident functions presented for SGLT2 inhibitors (purple) and control group (red). Shaded areas are 95% confidence intervals.

**Fig. 3:**
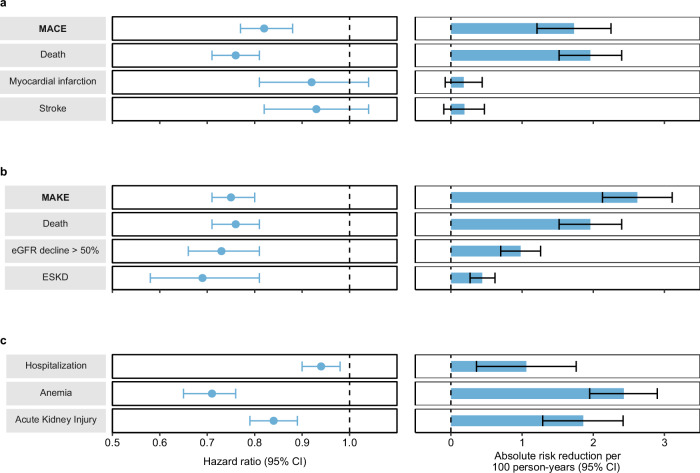
Hazard ratio and absolute risk reduction of the primary and secondary outcomes in the SGLT2 inhibitors vs the control group. n for SGLT2 inhibitors = 11,588, n for control group = 96,188. **a** Major adverse cardiovascular events (MACE) and its components including death, myocardial infarction and stroke; **b** Major adverse kidney events (MAKE) and its components including death, eGFR decline > 50%, and end stage kidney disease; **c** secondary outcomes including hospitalization, anemia and acute kidney injury. Adjusted hazard ratios and 95% confidence intervals are presented. Length of the bar represents the risk reduction per 100 persons at 180 days and associated 95% confidence intervals are also shown.

Over the follow up period (median 1.57 (IQR: 1.05–2.49) years) which corresponded to 184,563 person-years of follow up, compared to the control group, SGLT2 inhibitors use was associated with reduced risk MACE (HR 0.82 (0.77, 0.88), ARR 1.73 (1.21, 2.25)); the point estimates for the individual components of MACE were HR 0.76 (0.71, 0.81), ARR 1.96 (1.52, 2.40) for death, HR 0.92 (0.81, 1.04). ARR 0.18 (−0.08, 0.44) for myocardial infarction, and HR 0.93 (0.82, 1.04), ARR 0.19 (−0.10, 0.47) for stroke (Figs. 2, 3, Supplementary Table 2).

Compared to the control group, SGLT2 inhibitors use was associated with reduced risk of MAKE (HR 0.75 (0.71, 0.80), ARR 2.62 (2.13, 3.11)). SGLT2 inhibitors use was associated with reduced risk of the individual components of MAKE including death (HR 0.76 (0.71, 0.81), ARR 1.96 (1.52, 2.40)), eGFR decline > 50% (HR 0.73 (0.66, 0.81), ARR 0.98 (0.70, 1.26)), and ESKD (HR 0.69 (0.58, 0.81), ARR 0.44 (0.27, 0.62)) (Figs. 2, 3, Supplementary Table 3).

**Fig. 4:**
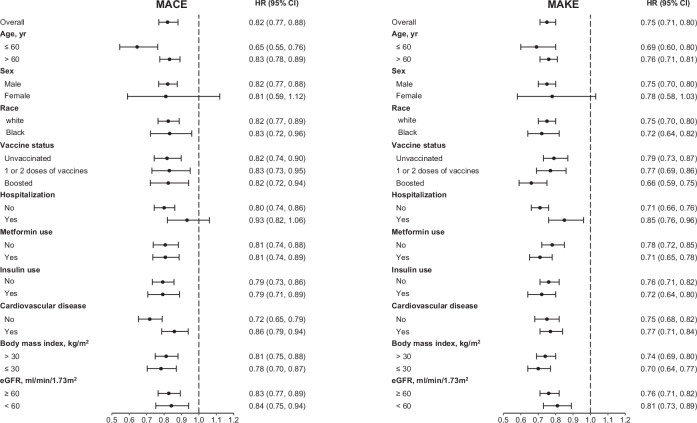
Risks major adverse cardiovascular events (MACE) and major adverse kidney events (MAKE) in the SGLT2 inhibitors vs the control group by subgroups. n for SGLT2 inhibitors=11,588, n for control group= 96,188. Subgroups including age ( ≤ 60 (n for SGLT2 inhibitor = 2822, n for control group = 21,867) and > 60 years (n for SGLT2 inhibitor = 8766, n for control group = 74,321)), sex (male (n for SGLT2 inhibitor = 11,024, n for control group = 89,593) and female (n for SGLT2 inhibitor = 564, n for control group = 6595), race (white (n for SGLT2 inhibitor = 8457, n for control group = 70,088 and Black (n for SGLT2 inhibitor = 2368, n for control group = 20,403), vaccination status (unvaccinated (n for SGLT2 inhibitor = 4026, n for control group = 45,887), received 1 or 2 doses of vaccine (n for SGLT2 inhibitor = 3382, n for control group = 25,271), boosted (n for SGLT2 inhibitor = 4180, n for control group = 25,030), status of hospitalization during acute phase of infection (n for SGLT2 inhibitor = 1759, n for control group = 14,725), status of non-hospitalization during acute phase of infection (n for SGLT2 inhibitor = 9829, n for control group = 81,463), metformin use (n for SGLT2 inhibitor = 5500, n for control group = 64,892), no metformin use (n for SGLT2 inhibitor =6088, n for control group =31,296), insulin use (n for SGLT2 inhibitor = 3499, n for control group = 30,825), no insulin use (n for SGLT2 inhibitor =8089, n for control group = 65,363), cardiovascular disease (n for SGLT2 inhibitor = 5999, n for control group = 35,418), no cardiovascular disease (n for SGLT2 inhibitor =5589, n for control group = 60,770), BMI ( > 30 (n for SGLT2 inhibitor =7496, n for control group = 61,396) and ≤ 30 kg/m^2^ (n for SGLT2 inhibitor = 4092, n for control group = 34,792)) and eGFR ( ≥ 60 (n for SGLT2 inhibitor =7929, n for control group = 71,288) and < 60 ml/min/1.73m^2^ (n for SGLT2 inhibitor =3659, n for control group = 24,900)).

Compared to the control group, SGLT2 inhibitors was associated with reduced risk of the secondary outcomes of hospitalization (HR 0.94 (0.90, 0.98), ARR 1.06 (1.36, 1.76)) anemia (HR 0.71 (0.65, 0.76), ARR 2.43 (1.95, 2.90)), and AKI (HR 0.84 (0.79, 0.89), ARR 1.86 (1.29, 2.42)) (Fig. 3, Supplementary Table 4). SGLT2 inhibitors use was associated with reduced risk of the secondary outcome of hospitalization (HR 0.91 (0.84, 0.98)) during the acute phase (first 30 days) of SARS-CoV-2 infection. We examined the association between SGLT2 inhibitors use and the risks of MACE and MAKE in several subgroups including age ( ≤ 60 and > 60 years), sex (male and female), race (white and Black), vaccination status (unvaccinated, received 1 or 2 doses of vaccine, boosted), status of hospitalization during acute phase of SARS-CoV-2 infection, metformin use, insulin use, cardiovascular disease status, BMI ( > 30 and ≤ 30 kg/m^2^) and eGFR ( ≥ 60 and < 60 ml/min/1.73m^2^). Compared to the control group, SGLT2 inhibitors use was associated with reduced risk of the composite outcomes of MACE and MAKE in most subgroups (Fig. 4, Supplementary Tables 5–6).

### Sensitivity analyses

Multiple sensitivity analyses were conducted to assess the robustness of our findings. (1) We applied an overlap weighting method instead of the inverse probability weighting method used in the primary approach; (2) we used doubly robust adjustment for covariates in the weighted survival model, instead of solely balancing based on the weighting in the primary approach; (3) we redefined the exposure group as those who initiated SGLT2 inhibitors within 180 days and, separately, within 90 days before SARS-CoV-2 infection, to proxy incident use instead of defining the exposure group as those who initiated SGLT2 inhibitors within 1 year before infection as in the primary approach; (4) we additionally adjusted for healthcare utilization factors such as the number of outpatient and inpatient visits, the number of laboratory tests, and the number of prescriptions received during follow-up, instead of only adjusting for baseline characteristics as in the primary approach. (5) We additionally adjusted for time-varying HbA1c values during follow-up, instead of only adjusting for baseline HbA1c as in the primary approach; (6) We conducted per-protocol analyses based on inverse probability of censoring weight where the protocol for the SGLT2 inhibitors group was defined as continued use of the SGLT2 inhibitors during follow up and the protocol for the control group was defined as non-use SGLT2 inhibitors during follow up, whereas intention to treat analyses were used in the primary approach. Results from all sensitivity analyses were consistent with our main findings (Supplementary Table 7).

## Discussion

In this study, we enrolled 107,776 people with SARS-CoV-2 infection — including 11,588 users of SGLT2 inhibitors and 96,188 users of other antihyperglycemics — and followed them for a median of 1.57 (IQR: 1.05–2.49) years after infection which altogether corresponded to 184,563 person-years of follow up. Compared to the control group, use SGT2 inhibitors was associated with reduced risk of MACE and MAKE. SGLT2 inhibitors use was also associated with reduced risk of hospitalization, anemia and AKI. Altogether, the findings that suggest that among people on antihyperglycemic therapy who contract SARS-CoV-2 infection, use of SGLT2 inhibitors was associated with cardiovascular and kidney protective effects. These findings may help inform choice of antihyperglycemic therapy.

People with diabetes have increased risk of adverse cardiovascular and kidney events^6,8^. SARS-CoV-2 itself is associated with increased risks of diabetes^26,40^, cardiovascular and kidney events for at least a year after SARS-CoV-2 infection and reinfection^12,26,29,31,40–42^. Evidence also suggests that the adverse health effects of SARS-CoV-2 may be even more pronounced in people with comorbidities including diabetes^29^. Our results may help aid in management decisions and choice of antihyperglycemic therapy to maintain cardiovascular and kidney health in people with both diabetes and SARS-CoV-2.

Both the DARE-19 and the RECOVERY trial were built on the hypothesis that SGLT2 inhibitors may reduce the risk of acute adverse health outcomes in people hospitalized for SARS-CoV-2 infection^10,11^. Both showed non-statistically significant results. Both trials focused exclusively on hospitalized individuals and only examined acute outcomes (within 28 days). Our subgroup analyses according to hospitalization status during the acute phase of the infection show that the salutary association of SGLT2 inhibitors with both MACE and MAKE was weaker among those hospitalized than non-hospitalized – which may explain the results of these two trials.

Our results are consistent with the large body of evidence showing protective cardiac and kidney benefits of SGLT2 inhibitors in people who require antihyperglycemic therapy^8^. Evidence suggests that SGLT2 inhibitors may provide protective cardiorenal effects through various mechanisms beyond glucose control^43–45^. SGLT2 inhibitors also reduce the risk of acute kidney disease; whether and to what extent the reduction in risks of MACE and MAKE is mediated by reduction in risk of acute kidney disease (a risk factor for both MACE and MAKE) should be evaluated in future studies. However, the question of whether those for whom antihyperglycemic therapy may not be indicated would benefit from initiation of SGLT2 inhibitors remains to be addressed (e.g., whether those at high risk of cardiovascular and kidney events following SARS-CoV-2 infection (sans diabetes and other established indications for SGLT2 inhibitors) may derive benefit from initiation of SGLT2 inhibitors to lessen the risk of cardiovascular and kidney disease post-COVID is yet to be investigated)^46–48^.

This study also has strengths. It was conducted using real-world data from the VA and incorporated information across multiple data domains, including demographics, diagnoses, laboratory tests, medications, vital signs, healthcare utilization, and contextual factors. The study was conducted within the VA, which provides prescription benefits to study participants, thereby reducing biases related to financial considerations (i.e. cost of SGLT2 inhibitors). We examined and reported the risk of MACE and MAKE on both the relative and absolute scales – the latter provides quantitative estimates of risk reduction on the absolute scale which may help decision-making by patients, healthcare providers and policy makers. The robustness of our approach was assessed through multiple sensitivity analyses, which yielded consistent results.

This study also has several limitations. The VA population is predominantly white and male, which may limit the generalizability of our findings. We evaluated effectiveness within those who had a positive SARS-CoV-2 test result; our study may not represent those infected but were not tested for SARS-CoV-2. Although we carefully designed our study and balanced characteristics across multiple data domains, biases including residual confounding and misclassification may not be ruled out. We relied on VA pharmacy records to define exposure. If participants in the control group received SGLT2 inhibitors outside of the VA, the observed difference between the two groups might be biased toward null. Because initiation or switching of antihyperglycemics occur rather infrequently around the time of SARS-CoV-2, this precluded us from developing an incident user design where exposure would be defined as incident use of SGLT2 inhibitors or other antihyperglycemics at the time of infection; instead, we evaluated the effect of current use of SGLT2 inhibitors within those who initiated this treatment within one year of SARS-CoV-2 infection. We focused on the outcomes of MACE and MAKE and did not explore the association between SGLT2 inhibitors and other adverse outcomes of COVID-19. We only examined the effect of the SGLT2 inhibitor class and did not evaluate the effect of each medication within this drug class. Different types of SGLT2 inhibitors may have different effects on the examined outcomes^49^. We evaluated the effect of SGLT2 inhibitors in people with SARS-CoV-2; our cohorts did not include a control group of participants without SARS-CoV-2 infection; consequently, we do not disentangle the effect of SGLT2 inhibitors on outcomes that are caused by SARS-CoV-2 from those caused by other pathways^50^. Due to the dynamic nature of the pandemic, including the mutation of SARS-CoV-2, changes in immunity levels in the population, and alterations in treatment plans for COVID-19, the underlying risk of the population may change and as a result, the effectiveness of SGLT2 inhibitors on risks of MACE and MAKE in people with SARS-CoV-2 infection may also change over time^51^.

In sum, among people with SARS-CoV-2 infection on antihyperglycemic therapy, those on SGLT2 inhibitors had less risk of MACE and MAKE and several secondary endpoints. These results suggest that SGLT2 inhibitors maintain their cardiovascular and kidney protective effects in people with SARS-CoV-2 infection. The findings may help guide use of antihyperglycemic therapy in people with SARS-CoV-2 infection.

## Supplementary information

Supplementary Information

Description of Additional Supplementary Files

Supplementary Data 1

Supplementary Data 2

Reporting Summary

## Data Availability

The data that support the findings of this study are available from the US Department of Veterans Affairs. Data from the US Department of Veterans Affairs must be securely stored behind VA firewall and only investigators approved by the VA could have access to the data. VA data are made freely available to researchers behind the VA firewall with an approved VA study protocol. For more information, please visit https://www.virec.research.va.gov or contact the VA Information Resource Center (VIReC) at VIReC@va.gov. The numerical data (source data) underlying Fig. 2 can be found in Supplementary Tables 2 and 3. The numerical data (source data) underlying Fig. 3 can be found in Supplementary Tables 2–4. The numerical data (source data) underlying Fig. 4 can be found in Supplementary Tables 2, 3, 5, and 6.
