# Supplementary material for: Adverse cardiovascular and kidney outcomes in people with SARS-CoV-2 treated with SGLT2 inhibitors
Source: Commun Med (Lond). 2024 Sep 11;4:179. doi: 10.1038/s43856-024-00599-4 (PMC11391050; doi:10.1038/s43856-024-00599-4)
Supplement: Supplementary file 1 — Supplementary Information [file 43856_2024_599_MOESM1_ESM.pdf]

Adverse cardiovascular and kidney outcomes in people with SARS-CoV-2 treated with SGLT2 inhibitors

Taeyoung Choi; Yan Xie; Ziyad Al-Aly

Table of Contents

Supplementary Figure 1. Covariate balance between SGLT2 inhibitors and the control groups 2
Supplementary Table 1. Outcome definition 3
Supplementary Table 2. Risk of MACE and its components in the SGLT2 inhibitors vs the control group 4
Supplementary Table 3. Risk of MAKE and its components in the SGLT2 inhibitors vs the control group 5
Supplementary Table 4. Risk of secondary outcomes in the SGLT2 inhibitors vs the control group 6
Supplementary Table 5. Subgroup analyses of the risk of MACE in the SGLT2 inhibitors vs the control group .. 7
Supplementary Table 6. Subgroup analyses of the risk of MAKE in the SGLT2 inhibitors vs the control group .. 8
Supplementary Table 7. Sensitivity analyses 9

Supplementary Figure 1. Covariate balance between SGLT2 inhibitors and the control groups

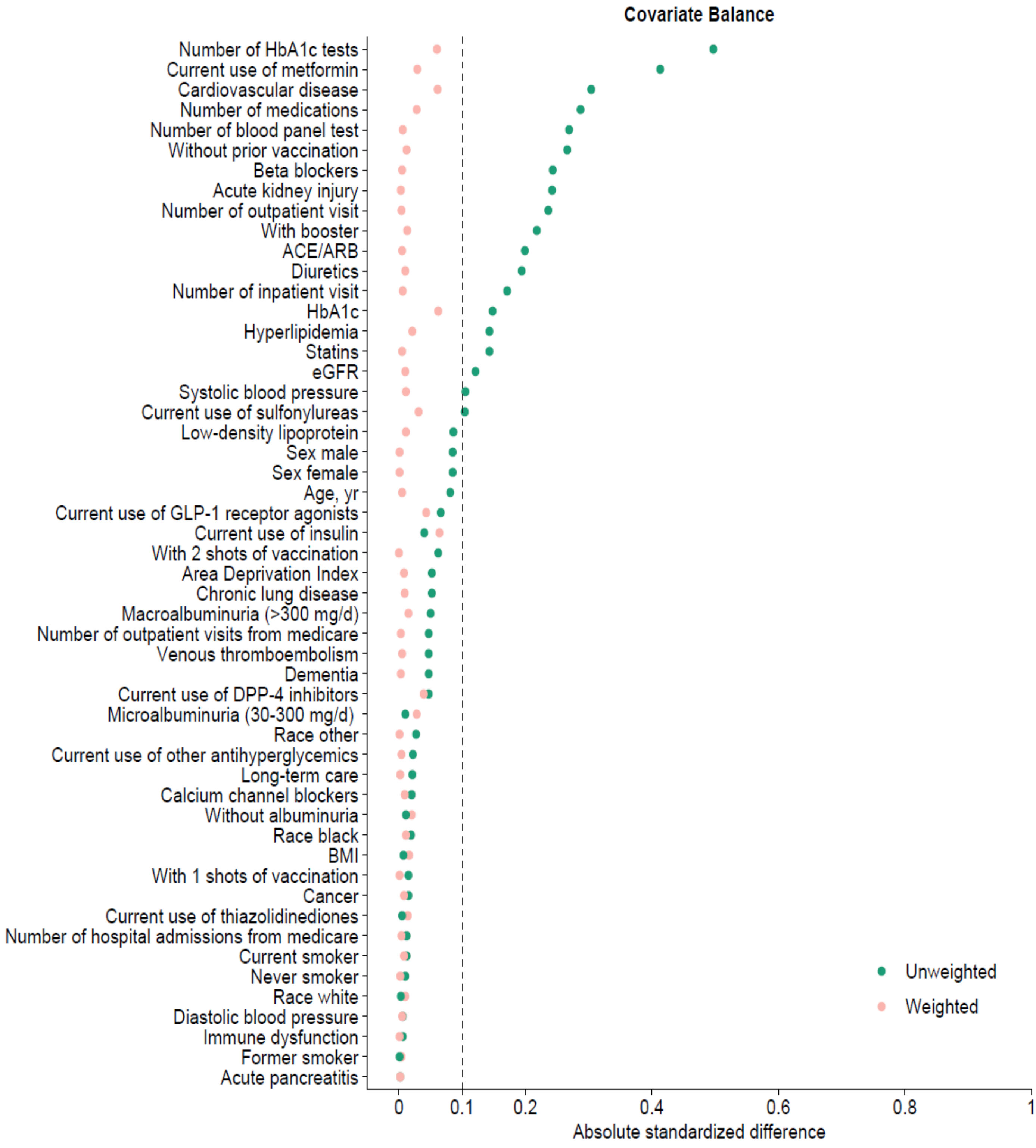

Supplementary Table 1. Outcome definition

	Outcomes	Definition
MACE	Myocardial Infarction	ICD10 code: I21, I22
	Stroke	ICD10 code: I63, I64, I69
MAKE	eGFR decline >50%	eGFR computed based on CKD-EPI without race formula
	ESKD	eGFR < 15 ml/min/1.73m^2 or dialysis or kidney transplant
Others	Anemia	ICD10 code: D60, D61, D62, D63, D64
	Acute Kidney Injury	50% or 0.3 mg/dl increase in serum creatinine within 90 days during follow up

MACE (major adverse cardiovascular events) was a composite of death, myocardial infarction and stroke.
MAKE (major adverse kidney events) was a composite of death, eGFR decline > 50%, and ESKD.
ESKD, end stage kidney disease

Supplementary Table 2. Risk of MACE and its components in the SGLT2 inhibitors vs the control group

Outcome	Hazard Ratio (95% CI)	SGLT2 inhibitors group event rate per 100 person-years (95% CI)	Control group event rate per 100 person-years (95% CI)	Absolute risk reduction per 100 person-years
MACE	0.82 (0.77, 0.88)	8.43 (7.93, 8.92)	10.16 (9.95, 10.36)	1.73 (1.21, 2.25)
Death	0.76 (0.71, 0.81)	6.48 (6.07, 6.90)	8.44 (8.26, 8.62)	1.96 (1.52, 2.40)
Myocardial infarction	0.92 (0.81, 1.04)	2.09 (1.85, 2.34)	2.27 (2.17, 2.38)	0.18 (-0.08, 0.44)
Stroke	0.93 (0.82, 1.04)	2.42 (2.15, 2.69)	2.61 (2.49, 2.72)	0.19 (-0.10, 0.47)

MACE (major adverse cardiovascular events) was a composite of death, myocardial infarction and stroke.
Outcomes were ascertained from the SARS-CoV-2 positive test date until the end of follow-up.
CI, confidence interval

Supplementary Table 3. Risk of MAKE and its components in the SGLT2 inhibitors vs the control group

Outcome	Hazard Ratio (95% CI)	SGLT2 inhibitors group event rate per 100 person-years (95% CI)	Control group event rate per 100 person-years (95% CI)	Absolute risk reduction per 100 person-years
MAKE	0.75 (0.71, 0.80)	8.43 (7.96, 8.89)	11.04 (10.84, 11.25)	2.62 (2.13, 3.11)
Death	0.76 (0.71, 0.81)	6.48 (6.07, 6.90)	8.44 (8.26, 8.62)	1.96 (1.52, 2.40)
eGFR decline > 50%	0.73 (0.66, 0.81)	2.75 (2.48, 3.02)	3.73 (3.60, 3.86)	0.98 (0.70, 1.26)
ESKD	0.69 (0.58, 0.81)	0.98 (0.82, 1.14)	1.43 (1.35, 1.51)	0.44 (0.27, 0.62)

MAKE (major adverse kidney events) was a composite of death, eGFR decline > 50%, and ESKD.
Outcomes were ascertained from the SARS-CoV-2 positive test date until the end of follow-up.
CI, confidence interval; ESKD, end stage kidney disease

Supplementary Table 4. Risk of secondary outcomes in the SGLT2 inhibitors vs the control group

Outcome	Hazard Ratio (95% CI)	SGLT2 inhibitors group event rate per 100 person-years (95% CI)	Control group event rate per 100 person-years (95% CI)	Absolute risk reduction per 100 person-years
Hospitalization	0.94 (0.90, 0.98)	17.56 (16.89, 18.23)	18.63 (18.37, 18.89)	1.06 (0.36, 1.76)
Anemia	0.71 (0.65, 0.76)	6.07 (5.63, 6.52)	8.50 (8.29, 8.70)	2.43 (1.95, 2.90)
Acute Kidney injury	0.84 (0.79, 0.89)	10.34 (9.80, 10.87)	12.19 (11.97, 12.41)	1.86 (1.29, 2.42)
Outcomes were ascertained from the SARS-CoV-2 positive test date until the end of follow-up.<br>CI, confidence interval				

Supplementary Table 5. Subgroup analyses of the risk of MACE in the SGLT2 inhibitors vs the control group

Subgroup	Description	Hazard Ratio (95% CI)	SGLT2 inhibitors group event rate per 100 person-years (95% CI)	Control group event rate per 100 person-years (95% CI)	Absolute risk reduction per 100 person-years
Age, yr	≤ 60	0.65 (0.55, 0.76)	3.70 (3.10, 4.30)	5.68 (5.36, 6.00)	1.98 (1.33, 2.62)
	> 60	0.83 (0.78, 0.89)	9.55 (8.93, 10.16)	11.37 (11.12, 11.62)	1.82 (1.18, 2.46)
Sex	Male	0.82 (0.77, 0.88)	8.48 (7.96, 8.99)	10.23 (10.01, 10.44)	1.75 (1.21, 2.29)
	Female	0.81 (0.59, 1.12)	6.53 (4.51, 8.51)	8.01 (7.32, 8.70)	1.48 (-0.59, 3.56)
Race	white	0.82 (0.77, 0.89)	8.43 (7.86, 9.01)	10.14 (9.90, 10.38)	1.71 (1.10, 2.32)
	Black	0.83 (0.72, 0.96)	8.56 (7.43, 9.68)	10.20 (9.75, 10.65)	1.64 (0.46, 2.82)
Vaccine	Unvaccinated	0.82 (0.74, 0.90)	10.98 (10.02, 11.93)	13.27 (12.94, 13.60)	2.29 (1.29, 3.28)
	1 or 2 doses of vaccine	0.83 (0.73, 0.95)	7.20 (6.33, 8.06)	8.57 (8.20, 8.95)	1.37 (0.46, 2.29)
	Boosted	0.82 (0.72, 0.94)	6.95 (6.10, 7.80)	8.37 (7.98, 8.76)	1.42 (0.51, 2.34)
Hospitalization	No	0.80 (0.74, 0.86)	7.00 (6.51, 7.48)	8.67 (8.46, 8.88)	1.67 (1.16, 2.18)
	Yes	0.93 (0.82, 1.06)	16.52 (14.63, 18.37)	17.62 (16.93, 18.29)	1.10 (-0.86, 3.05)
Metformin use	No	0.81 (0.74, 0.88)	8.84 (8.12, 9.56)	10.84 (10.47, 11.21)	2.00 (1.22, 2.78)
	Yes	0.81 (0.74, 0.89)	7.75 (7.07, 8.43)	9.51 (9.27, 9.75)	1.76 (1.05, 2.47)
Insulin use	No	0.79 (0.73, 0.86)	7.69 (7.13, 8.25)	9.61 (9.37, 9.85)	1.91 (1.32, 2.51)
	Yes	0.79 (0.71, 0.89)	8.66 (7.73, 9.57)	10.79 (10.41, 11.17)	2.14 (1.17, 3.11)
Cardiovascular disease	No	0.72 (0.65, 0.79)	6.83 (6.21, 7.43)	9.38 (9.14, 9.62)	2.56 (1.91, 3.20)
	Yes	0.86 (0.79, 0.94)	9.84 (9.04, 10.62)	11.36 (10.97, 11.74)	1.52 (0.68, 2.36)
Body mass index, kg/m^2	> 30	0.81 (0.75, 0.88)	7.93 (7.34, 8.53)	9.68 (9.43, 9.93)	1.75 (1.12, 2.37)
	≤ 30	0.78 (0.70, 0.87)	8.95 (8.07, 9.83)	11.30 (10.93, 11.66)	2.35 (1.42, 3.27)
eGFR, $ml/min/1.73m^2$	≥ 60	0.83 (0.77, 0.89)	8.15 (7.56, 8.73)	9.77 (9.53, 10.00)	1.62 (1.01, 2.23)
	< 60	0.84 (0.75, 0.94)	9.50 (8.52, 10.46)	11.18 (10.75, 11.61)	1.68 (0.66, 2.71)

CI, confidence interval

Supplementary Table 6. Subgroup analyses of the risk of MAKE in the SGLT2 inhibitors vs the control group

Subgroup	Description	Hazard Ratio (95% CI)	SGLT2 inhibitors group event rate per 100 person-years (95% CI)	Control group event rate per 100 person-years (95% CI)	Absolute risk reduction per 100 person-years
Age, yr	≤ 60	0.69 (0.60, 0.80)	4.96 (4.27, 5.64)	7.10 (6.75, 7.45)	2.14 (1.41, 2.88)
	> 60	0.76 (0.71, 0.81)	9.30 (8.74, 9.86)	12.12 (11.88, 12.37)	2.82 (2.23, 3.42)
Sex	Male	0.75 (0.70, 0.80)	8.43 (7.96, 8.91)	11.10 (10.89, 11.31)	2.67 (2.16, 3.17)
	Female	0.78 (0.58, 1.03)	7.69 (5.63, 9.71)	9.81 (9.05, 10.56)	2.11 (-0.01, 4.24)
Race	white	0.75 (0.70, 0.80)	8.35 (7.81, 8.88)	10.97 (10.73, 11.21)	2.63 (2.06, 3.20)
	Black	0.72 (0.64, 0.82)	8.65 (7.60, 9.69)	11.76 (11.30, 12.22)	3.11 (2.00, 4.21)
Vaccine	Unvaccinated	0.79 (0.73, 0.87)	10.43 (9.55, 11.30)	12.94 (12.62, 13.26)	2.51 (1.60, 3.42)
	1 or 2 doses of vaccine	0.77 (0.69, 0.86)	8.15 (7.28, 9.01)	10.45 (10.06, 10.85)	2.30 (1.38, 3.23)
	Boosted	0.66 (0.59, 0.75)	6.16 (5.45, 6.87)	9.14 (8.76, 9.53)	2.98 (2.20, 3.76)
Hospitalization	No	0.71 (0.66, 0.76)	6.79 (6.35, 7.24)	9.48 (9.27, 9.69)	2.69 (2.21, 3.16)
	Yes	0.85 (0.76, 0.96)	15.62 (13.98, 17.23)	18.07 (17.43, 18.71)	2.45 (0.74, 4.16)
Metformin use	No	0.78 (0.72, 0.85)	8.97 (8.30, 9.63)	11.30 (10.93, 11.66)	2.33 (1.61, 3.05)
	Yes	0.71 (0.65, 0.78)	7.55 (6.92, 8.19)	10.40 (10.16, 10.65)	2.85 (2.19, 3.51)
Insulin use	No	0.76 (0.71, 0.82)	7.88 (7.35, 8.41)	10.20 (9.96, 10.44)	2.32 (1.76, 2.89)
	Yes	0.72 (0.64, 0.80)	8.27 (7.44, 9.10)	11.37 (11.00, 11.74)	3.10 (2.21, 3.98)
Cardiovascular disease	No	0.75 (0.68, 0.82)	6.73 (6.14, 7.30)	8.87 (8.64, 9.11)	2.15 (1.54, 2.75)
	Yes	0.77 (0.71, 0.84)	9.11 (8.44, 9.78)	11.66 (11.32, 12.01)	2.55 (1.83, 3.27)
Body mass index, kg/m^2	> 30	0.74 (0.69, 0.80)	7.74 (7.19, 8.29)	10.28 (10.03, 10.53)	2.54 (1.95, 3.12)
	≤ 30	0.70 (0.64, 0.77)	8.75 (7.95, 9.55)	12.25 (11.89, 12.61)	3.50 (2.65, 4.34)
eGFR, $ml/min/1.73m^2$	≥ 60	0.76 (0.71, 0.82)	8.15 (7.59, 8.70)	10.55 (10.32, 10.78)	2.40 (1.82, 2.99)
	< 60	0.81 (0.73, 0.89)	9.90 (9.00, 10.79)	12.11 (11.69, 12.53)	2.21 (1.26, 3.16)
CI, confidence interval					

Supplementary Table 7. Sensitivity analyses

Sensitivity analyses	MACE HR (95% CI)	MAKE HR (95% CI)
Applied overlap weighting method	0.83 (0.78, 0.88)	0.74 (0.70, 0.79)
Applied doubly robust method	0.84 (0.79, 0.90)	0.76 (0.72, 0.81)
Initiated SGLT2 inhibitors within 180 days before SARS-CoV-2 infection	0.85 (0.79, 0.92)	0.76 (0.71, 0.82)
Initiated SGLT2 inhibitors within 90 days before SARS-CoV-2 infection	0.85 (0.76, 0.94)	0.77 (0.70, 0.85)
Adjusting for health care utilization during the follow up	0.81 (0.76, 0.87)	0.76 (0.72, 0.81)
Adjusting for HbA1c during the follow up	0.80 (0.75, 0.86)	0.75 (0.70, 0.79)
Per-Protocol analyses	0.73 (0.67, 0.81)	0.74 (0.68, 0.80)
MACE (major adverse cardiovascular events) was a composite of death, myocardial infarction and stroke.<br>MAKE (major adverse kidney events) was a composite of death, eGFR decline > 50%, and ESKD.<br>CI, confidence interval; ESKD, end stage kidney disease		
