# Supplementary material for: Adverse cardiovascular and kidney outcomes in people with SARS-CoV-2 treated with SGLT2 inhibitors
Source: Commun Med (Lond). 2024 Sep 11;4:179. doi: 10.1038/s43856-024-00599-4 (PMC11391050; doi:10.1038/s43856-024-00599-4)
Supplement: Supplementary file 2 — Description of Additional Supplementary Files [file 43856_2024_599_MOESM2_ESM.pdf]

Description of Additional Supplementary Files

File name- Supplementary Data 1

File description- Demographic and health characteristics of the cohorts before weighting

File name- Supplementary Data 2

File description- Demographic and health characteristics of the cohorts after weighting
